# Supplementary material for: Are carbon emissions trading and green financial instruments synergistic? -Comprehensive quantitative research based on content analysis
Source: PLoS One. 2024 Mar 7;19(3):e0298601. doi: 10.1371/journal.pone.0298601 (PMC10919601; doi:10.1371/journal.pone.0298601)
Supplement: S1 Data — (ZIP) [file pone.0298601.s001.zip › Data/地级市Z_F工作报告.zip!/╜¡╦╒/┴1⁄4╘╞╕█╩╨/└┤╫╘╣1⁄2╓┌║┼í╛┬φ┐╦╩2╛▌═°í┐_2003.docx]

各位代表：

　　现在，我代表市人民政府向大会作工作报告，请予审议，并请各位政协委员和其他列席人员提出意见。

　　　　　　　　　　　　　　　　　　　　　　　　　　　　　　　　　　　　　　　　过去五年的工作回顾

　　市十届人民代表大会第一次会议以来的五年，是我市跨世纪发展的重要时期。在中共连云港市委的领导下，全市人民高举邓小平理论伟大旗帜，认真实践"三个代表"重要思想，解放思想，实事求是，攻坚克难，锐意进取，经济建设和社会发展取得显著成绩。

　　一、结构调整成效明显，经济实力逐步增强

　　五年来，全市国民经济在调整中发展、在优化中提高。国内生产总值年均递增9.3％，其中一二三产分别递增3.5％、12.8％、8.7％，财政收入年均递增12.4％。三次产业比例由1997年的32.5∶37.8∶29.7调整为23.4∶44.3∶32.3。2002年经济增长创近几年最好水平，国内生产总值达350.3亿元，增长11.4％；财政收入26.98亿元，增长31.2％，剔除新纳入预算管理的行政性收费和政府性基金，增长11.7％，其中市本级增长14％；全社会固定资产投资175亿元，增长15.3％；直接利用外资1.1亿美元，增长93.3％。除外贸出口增长2.5％未达计划要求外，其他各项主要经济指标都完成或超额完成预期目标。

　　大力发展高效农业、外向型农业，农业和农村经济克服特大水灾等灾害的影响，取得全面发展。粮经产值比由1997年的5.5∶4.5调整为2002年的3.8∶6.2。林牧渔业不断壮大，林业、牧业产值分别年均递增8.4％和4％，海洋经济规模化程度不断提高，建成全省最大的海水育苗基地，海洋产业总产值年均递增20％。产业化经营快速推进，重点龙头企业发展到109家，形成特色乡镇59个，建成蔬菜、紫菜等十大农产品出口创汇基地，新创农产品品牌51个。农机跨区作业连续四年被评为全国先进。

　　工业经济主体地位初步确立，发展后劲不断增强。工业增加值占GDP比重由32.6％提高到36.3％，完成工业技术改造投资186亿元，高新技术产业产值年均递增20％以上。年销售收入过亿元重点骨干企业增加16户，医药、纺织、化工等五个主要行业占工业增加值比重提高7.2个百分点，经济集中度明显提高。乡镇工业小区发展较快，涌现出一批规模龙头加工企业。2002年工业经济增速省内领先，工业增加值增长14.3％。建筑业快速发展，国内外建筑市场不断巩固扩大，增加值占GDP比重由5.2％增加到8％。

　　商贸流通、交通运输等传统服务业稳步发展，旅游、房地产、社区服务等新兴服务业增势强劲。新型流通业态不断涌现，生产生活资料和要素市场发展较快，2002年完成社会消费品零售总额113亿元。市区公交线路由4条增加到25条，快速客运、零担运输等多样化运输服务迅速发展。港口生产连续跨越两个千万吨大关，2002年完成吞吐量3316万吨，集装箱20.5万标箱。连云港之夏、西游记文化旅游节影响不断扩大，游客接待量和旅游收入年均递增20％。2002年花果山景区和连岛度假区先后被评为国家AAAA级旅游区，并分别被评为全国文明风景旅游区示范点和全国健康型海水游泳场。市区拆除危旧房153万平方米，新增配套完善的住宅500多万平方米。社区建设初步形成市、区、街、居四级服务网络。法律、会计等中介服务业发展迅速。金融机构各项存款余额259.9亿元，各项贷款余额200.7亿元，比1998年初分别增加118.7亿元和51.6亿元。

　　二、各项改革取得突破，对外开放持续推进

　　五年来，我们坚持以改革总揽全局，以体制机制创新推进发展。国有企业改革强力推进，国有资产管理监督营运体系初步形成。组建7家股份有限公司，2家公司成功上市，国有大中型企业改制面达91％，78％的中小企业退出国有集体资本，乡镇企业改制基本完成，一批科技型外向型民营企业逐步发展壮大，非公有制经济实现增加值占GDP比重达42.1％。2002年市属企业改革力度进一步加大，80个改革发展重点项目全面完成，康缘药业成功上市，正大天晴、港口股份完成上市辅导，29户企业依法进入破产程序。投融资体制改革迈出坚实步伐，实施银企联席会议制度，市商业银行组建运营，农村信用社改革试点有效推进，金融与经济良性互动局面初步形成。保险业在改革中不断扩大。财税体制改革进一步深化，实施所得税收入分享改革，稳步推进预算编制改革，制定出台国库集中收付制度，全面实施预算外资金收支两条线管理，完善政府采购制度，财政运行机制逐步优化。城镇职工三项医疗制度和粮棉流通体制改革稳步推进，社会保障体系初步建立，全面推行住房分配货币化制度，城市管理体制和土地使用制度改革逐步推进。实施行政机构改革和区划调整，市县乡行政编制减少20％，市级行政机构减少13.6％，顺利撤并云台区和26个乡镇、654个村、74个居委会。在全省率先改革行政审批制度，市审批中心建立运行，市级审批项目减少573项。认真贯彻实施《农村土地承包法》，促进土地依法合理流转。农村税费改革和各项配套改革取得重要成果，取消和降低收费项目182项，农民负担人均减少101.8元，减负率达50.7％。

　　大力实施开放兴市战略，强力推进招商引资。五年实际利用外资12.6亿美元；2002年实际利用外资5.2亿美元，占五年的41.3％，益海粮油、罗盖特等一批投资规模大、经济效益好的外资企业投产达产或开工建设，杜钟氨纶、东海福润等企业进一步增资扩股。五年吸引内联到位资金32.6亿元。对外贸易持续增长，外贸出口年均递增23.8％。对外经济技术合作不断扩大，外经营业额年均递增13.5％。各级各类园区招商引资主阵地作用明显增强。开发区直接利用外资占全市45.3％，形成亚洲集装箱等一批骨干企业。医药、高新技术、民营经济等特色园区初具规模。2002年建成出口加工区一期工程，并具备封关运作条件。

　　三、基础设施日趋完善，城乡面貌明显改观

　　五年来，抓住国家扩大内需有利时机，大力加强基础设施建设。累计完成全社会固定资产投资647.2亿元，是前五年的2.5倍。实施了蔷薇河送清水、新沂河海口控制等一批重点水利工程和325平方公里山丘区小流域治理。建成高速公路213公里，改造国省道434公里，新建改建县乡灰黑化公路528公里、乡村砂石化公路1880公里，城乡交通条件大为改善。东陇海铁路复线和新火车站建成使用。港口基础设施建设加强，通过能力进一步提高。亚洲大陆第一口超深科学勘探井开钻。建成投产仓容4.05亿斤国家粮食储备库。信息网络基础设施快速发展，程控电话交换机容量突破90万门，移动电话交换机容量达76万门，城乡电话普及率达每百人20部。实施了信息港湾到县和企业、政府、家庭上网工程，宽带基础数据网和互联网已覆盖全市。城乡电网改造全面实施，建成一批大中型电力建设和改造项目，实现城乡居民用电同网同价。2002年42项重点建设项目完成年度投资98.6亿元，石梁河水库除险加固、大浦抽水站等重点水利工程建成，汾灌高速公路、连徐高速公路连云港段一期等投入使用，民航机场油库竣工，完成7万吨级深水航道疏浚工程，7-10万吨级深水泊位改造和庙岭三期集装箱顺岸泊位开工建设，田湾核电站一期工程进展顺利，1、2号机组主体设备安装调试，建成220千伏云台输变电工程。

　　高起点高标准编制市域城镇体系规划、修编城市总体规划，编制四县县城总体规划和小城镇总体规划，深化和完善城市专项规划，形成较为完善的规划编制体系，详细规划覆盖面不断扩大。城市建设总投资近100亿元，建成区面积由49.6扩大到61.1平方公里。

新建改建了60多条城市主次干道和一批重点市政工程，新增城市道路70多公里，城市人均公共绿地7.2平方米，绿化覆盖率33％，建成人防工程1.6万平方米。国展中心、新银双厦等标志性建筑相继建成，实施了新海污水处理和龙尾河、西盐河综合整治工程，新建了垃圾处理场扩容等一批环卫设施。新区建设全面启动，古城和海滨城建设取得明显成效。供热、供水、排水、燃气等公用事业迅速发展。花果山、连岛、孔望山等景区新增一批旅游项目，苏马湾、高公岛等相继建成开放。对重点地段、窗口单位和市民集中活动区域加强美化亮化，新增亮化面积18万平方米，环境综合整治不断加强，市容市貌和环境卫生质量大为改观。大力推进城乡一体化，城镇化率由24％提高到33％。四县县城建设步伐加快，实施了一批旧城改造、小区开发、道路建设工程，市政设施、科教文卫体等公共服务设施逐步完善，集聚规模和辐射功能进一步增强。一批富有特色的小城镇逐步崛起，创建4个省级示范小城镇和36个市级新型小城镇。2002年是城市基础设施大建设之年，市区完成七大类137个城建项目，总投资22.6亿元，项目数、投资额均创历史之最。原市政府地块等老城区改造项目顺利推进，新行政中心开工建设，瀛洲南路、凌洲广场、中山西路、新建东路等市政重点工程相继建成，一批与群众生活密切相关的道路和街巷得到建设改造。

　　四、科技教育快速发展，社会事业不断进步

　　五年来，大力推进科技进步，促进科技成果转化，提高产业技术水平。实施省以上火炬计划项目72项、星火计划项目144项，开发省以上高新技术产品179个，建成农业科技园45个，振兴现代农业科技示范园去年被批准为国家级示范园，引进推广农业、海洋新技术、新品种170余个。培育省高新技术企业21家、国家重点高新技术企业8家。2002年新医药产业基地成为国家级火炬产业基地，中电华威列为国家863计划成果产业化基地。建立企业工程中心、企业技术中心、博士后技术创新中心54家，恒瑞制药、康缘药业、716所3家国家级博士后科研工作站获得批准。工业、农业科技进步贡献率分别由23％、31.7％提高到37％、44.2％。大力发展教育事业，全面推进素质教育，两基成果进一步巩固。改造薄弱学校577所；消除危房72万平方米，于2002年提前一年完成省定目标。合理调整农村中小学布局，市区相继实施八校联动和十校重组。建成新海、赣榆、东海高级中学和灌南高级中学一期工程。4所高中创建为国家级示范性普通高中，14所中学创建为省重点中学，高中阶段教育入学率由45％提高到72％，5万多名学生被输送到各类高校。整合高等教育资源，组建了职业技术学院和师范专科学校，淮海工学院组合化工高专形成万人大学规模。重视人才资源开发，中专学历或初级职称以上人才总量由14万人扩大到19万人。

　　坚持以人为本，推进社会事业全面发展。文学艺术、新闻出版、广播影视事业健康活跃，一批优秀文艺作品获省以上奖项150多个，230件广播电视作品获国家、省级政府奖，文化市场健康有序发展。藤花落史前城址列入2000年度全国十大考古新发现，去年海州双龙汉墓女尸发掘轰动考古界。市县乡三级有线电视全部联网，行政村联网率达35％。市急救中心2002年竣工投入使用，60栋乡镇卫生院业务楼相继建成，医疗卫生条件进一步改善。全民健身运动蓬勃开展，获省以上竞技体育金牌176枚，成功举办第五届中国国际风筝会、市第十届运动会，基本建成省海上训练基地。人口自然增长率下降3个千分点，四县跨入省计划生育合格县行列。国土资源管理和土地复垦开发得到加强，实施采矿权招投标制度。环境质量得到有效保护，建成市区第一块清洁能源小区，空气质量和海洋气象情况在中央电视台播报。国防建设、民兵预备役和优抚安置工作得到加强，连续三次荣获全国双拥模范城称号。完成第五次人口普查和第二次基本单位普查。外事、侨务、对台事务、民族宗教、档案、地震、地方志工作取得新的成绩，妇女、儿童、老龄、关心下一代和残疾人事业得到重视和发展。

　　五、精神文明建设加强，民主法制进程加快

　　五年来，不断加强精神文明建设，城乡文明程度和市民文明素质进一步提高。广泛开展增强连云港意识、树立连云港人新形象大讨论，唱响热爱连云港、建设连云港、奉献连云港的主旋律，注重落实公民道德建设实施纲要，弘扬了新浦汽车总站雷锋车组等一大批富有时代精神的先进典型。以创建文明城市为龙头，整体推进文明村镇、文明社区、文明行业等精神文明创建活动，举办了三届在海一方广场文化系列活动和各类群众性文化艺术活动，我市被评为省创建文明城市工作先进市，青口镇等5个单位被评为全国精神文明创建工作先进单位。全市初步形成了经济建设和政治、文化建设相互促进、协调发展的良好态势。

　　各级政府自觉接受人大及其常委会的监督，坚持重大问题与人民政协协商，在工作中注意听取各民主党派、无党派人士、专家学者和人民群众的意见。加强基层民主政治建设，村民自治和社区居民自治有序推进。坚持依法治市，推进依法行政，完成三五普法任务，开展四五普法教育，基层法制工作不断加强。依法严厉打击各种犯罪活动，坚持打防结合、预防为主，全面落实社会治安综合治理各项措施。防范和惩治法轮功邪教组织及非法宗教活动。整顿和规范市场经济秩序。健全信访工作制度，落实信访责任制，妥善处理人民内部矛盾，有效维护了社会稳定。认真落实廉政建设责任制，查处违法违纪案件，开展纠风和专项治理，加强审计监督，各级政府组织、思想、作风建设得到加强。

　　六、人民生活得到改善，社会保障逐步加强

　　五年来，始终把提高人民生活水平作为政府工作的立足点和出发点，努力增加城乡居民收入，改善群众生产生活条件，人民生活在2000年总体达到小康水平基础上继续改善。2002年，城市居民人均可支配收入7628元，五年间平均递增7.6％；农民人均纯收入3000元，接近灾前1999年的水平，年均递增2.3％。人均储蓄由2022元提高到3443元，年均递增11.2％。居民消费结构明显改善，文化、教育、娱乐消费比例不断上升。归集城镇职工住房公积金6.4亿元，为城市中低收入家庭建设经济适用住房190万平方米，城镇居民人均住房使用面积由16.9平方米增加到20.9平方米，农村居民人均住房面积由20.9平方米增加到24平方米。农村降氟治污改水受益423个村84.2万人，传染病发病率低于全省平均水平。安全生产工作得到加强。

　　城镇职工社会保障体系初步建立，有效促进了劳动力资源的合理配置。大力推进两个确保和三条保障线的衔接，累计发放下岗职工基本生活保障金1.6亿元、养老金18.1亿元，1.5万城市贫困居民纳入最低生活保障范围。医疗、工伤、生育保险覆盖面不断扩大。通过实施农村扶贫、对口帮扶、实物帮困等措施，农村低收入和特困群众的基本生活得到保障，完成灾民建房任务，改造草危房162万平方米，救济灾民和困难群众176.3万人次，五保供养率达100％。广辟就业渠道，增加就业岗位，就业再就业工作稳步推进，5.4万名下岗职工实现再就业。多渠道转移农村富余劳动力，去年劳务输出总数达50万人，比上年增加10万人，成为农民增收的重要渠道。

　　各位代表，过去的五年，是我市综合实力明显增强、城乡面貌大为改观的五年，是市场取向改革取得突破、对外开放不断加快的五年，是社会事业协调发展、人民生活持续改善的五年。这些成就的取得，凝聚着全市人民的辛劳和智慧，是方方面面关心支持、共同奋斗的结果。在此，我代表市人民政府，向全市广大干部群众、驻连部队、武警官兵、公安干警、驻连部省属单位和支持连云港发展的海内外朋友，向各位人大代表、政协委员和各民主党派、各人民团体、各界友好人士，表示崇高的敬意和衷心的感谢！

　　在充分肯定成绩的同时，我们也清醒地看到，经济发展和社会生活中还存在着不少矛盾和困难：全市经济总量偏小，整体实力不强，市场化、工业化、城市化进程相对滞后；县域经济基础比较薄弱，财政收支矛盾突出；对外开放有待进一步拓展领域、提升层次、创新方式；农业抗灾能力较弱，农产品市场化程度偏低，农民持续增收任务艰巨；就业再就业压力较大，城乡特困群体的扶助措施需要有效落实；投资发展软环境必须继续改善，政府职能和干部作风仍需进一步转变。我们必须高度重视存在的问题，采取得力措施加以解决。

　　　　　　　　　　　　今后五年的展望

　　党的十六大确立了全面建设小康社会的奋斗目标，现代化建设事业进入了新的发展阶段。牢牢把握新世纪头二十年的重要战略机遇期，找准位置，发挥优势，我们必将大有作为。按照市委九届二次全会的部署，到2010年，全市国内生产总值在2000年基础上翻一番半，人均突破2000美元，建成宽裕型小康社会；再经过三五年努力，国内生产总值迈上千亿元台阶，人均突破3000美元，全面建成小康社会；到建党100周年时，力争国内生产总值在2010年的基础上再翻一番半，人均超过5000美元，总体上达到初步现代化水平。努力把连云港建设成为综合实力显著增强的新兴工业城市，全面融入经济全球化的国际性海港城市，环境优美、特色鲜明的中外知名旅游城市，港口、陆桥和区位优势得到充分发挥的区域性物流中心和商务中心。从现在起到2007年的新一届政府任期，是我市加快富民强市、全面建设小康社会的重要时期。我们将全面完成十五计划，制定实施新的五年计划，为实现全面建设小康社会目标打好基础。现就下届政府今后五年的工作目标提出如下建议。

　　今后五年，全市国民经济和社会发展主要预期调控目标为：国内生产总值年均递增11％左右，到2007年人均达到1400美元以上，初步进入工业化中期；财政收入高于GDP增长，到2007年突破50亿元；全社会固定资产投资累计突破1000亿元；实际利用外资累计20亿美元，外贸出口年均递增12％；2007年城镇化率达40％以上；城镇居民人均可支配收入年均递增8％；农民人均纯收入按照3＋2追赶计划，从今年起，经过3年努力，恢复到2000年灾前在苏北的位次，再通过2年左右的奋斗，力争达到苏北先进水平。

　　经过五年的奋斗，努力实现以下几个方面的突破：

　　经济发展实现新跨越。牢牢把握发展第一要务，尽快扩大经济总量，转变经济增长方式，营造区域发展比较优势，形成经济快速高效增长的良好局面。围绕农业增效农民增收，加快农业和农村经济结构调整，推进农业产业化、市场化、标准化和外向化，促进传统农业向现代农业转变。走新型工业化道路，以信息化带动工业化，大力发展新医药、新材料等高新技术产业，建成全国重要的新医药工业基地和重点新能源工业基地，形成高新技术产业和传统优势产业相融合的现代工业体系。大力发展以山海风光为特色的旅游业、以港口为依托的现代物流业，培育壮大信息、房地产、社区服务等新兴服务业。二三产业占GDP比重提高到85％以上。县域经济综合实力和发展竞争力进一步增强，主要指标在全省和苏北地区位次明显前移。

　　改革开放开创新局面。坚定推进市场取向的改革，继续大力实施开放兴市战略，全面提高经济的市场化程度和国际化水平。加快经济体制和运行机制创新，以产权制度改革为主线，全市国有经济比重降到25％左右，中小企业国有资本基本退出，尽快完善国有资产监管营运体系，初步形成资本市场的连云港药业板块，改革分配制度，建立有效的经营者激励约束机制。加快发展私营个体经济，深化投融资体制市场化改革，配套推进各项改革。发展和规范多层次的市场体系，加快审批型政府向服务型政府转变。进一步优化开放环境，吸引更多的外来投资，不断扩大商品和服务贸易，做强做大开放型经济这一龙头经济。开发区主要经济指标力争翻两番，在首批国家级开发区中位次前移，加速扩张其他各类经济园区。发挥国际性海港和东方桥头堡的优势，建成服务周边地区、辐射中西部的国际商务中心，进一步确立在苏北地区对外开放的优势地位。

　　特色经济形成新亮点。充分挖掘经济资源和发展潜力，着力培育壮大新的增长点，加快形成独具特色的产业优势，不断拓展经济增长空间，走差别竞争、特色取胜之路。强力推进以港兴市，大力发展港口经济。构建港口管理运作新模式，提高通过能力，拓展综合功能，2007年港口吞吐量突破5000万吨，集装箱突破50万标箱。加快发展临港工业、加工贸易和现代物流业。推动城市建设、产业布局和港口功能协调发展，促进港城一体化。合理开发和有效整合旅游资源，把连云港之夏、西游记文化旅游节办成旅游节庆精品，逐步壮大旅游产业。大力推进海上连云港建设，大力推进科技兴海，深度开发400万亩浅海域资源，积极发展海洋化工和海洋医药产业，加快建设赣榆海洋经济开发区，形成集渔港码头、加工贸易、养殖捕捞、观光旅游为一体的海洋经济发展高地。依托地方特色资源，通过外引内联提高硅资源开发水平，促进东海形成名符其实的水晶之都；发挥汤沟酒业、板浦滴醋等知名品牌优势，壮大特色食品加工业；逐步做大做强海产品、畜禽、林木等特色资源加工业。

　　城市发展塑造新形象。增强城市产业承载力和对外影响力，建设具有合理的城市布局、先进的交通网络、完备的配套设施、优良的城市秩序的现代化海港城市。依据城市总体规划，发掘城市文化内涵，做优做美中心城市，充分塑造山、水、港、城一体发展的城市个性，体现显山、露水、透绿、现蓝的城市特色，倾力打造名山名水、绿色生态、古城名郡、现代港城四大特色品牌，推进金镶玉竹美化景区、装点城市，形成名城名竹效应，彰显神奇、浪漫的城市风貌。新城区建设初具规模，完成老城区改造，构成东西城区同步发展、城区景区相互辉映、山水园林生态融为一体的组团式城市形态，努力创建适宜投资兴业和生活居住的中国优秀旅游城市、国家生态城市、国家环保模范城市。按照中心成网、外围成环、四周通达的格局，构建和完善城市综合交通体系，形成环城快速通道，建成连盐高速公路，建设沿海一级公路，成为全国公路主枢纽之一，力争实现东陇海铁路电气化。

　　小康生活迈入新阶段。以全面建设小康社会为奋斗目标，大力推进科教兴市和可持续发展战略，协调发展经济、科技教育和各项社会事业，全面改善群众物质文化生活，基本实现富裕型小康。人均GDP达12000元以上，超过全国平均水平。城镇居民可支配收入达到10000元，农民人均纯收入达到4500元以上。人均储蓄年均增长12％以上。城乡居民人均住房面积分别增加7和5平方米，恩格尔系数下降4个百分点。社会保障体系比较健全，社会就业相对充分，全民思想道德素质、科学文化素质和健康素质明显提高，人均受教育年限由9.4年提高到11.5年，逐步建成学习型城市，促进人的全面发展。建设博物馆、档案馆、体育中心、文化艺术中心等标志性文体工程。认真贯彻公民道德建设实施纲要，加快推进与市场经济相适应、与法律规范相协调、与传统美德相承接的公民思想道德体系建设。社会主义民主更加完善、法制更加完备，依法治市得到全面落实，社会诚信体系逐步确立。生态环境得到保护，资源利用率显著提高，人与自然较为和谐。通过五年的努力，进一步提升社会凝聚力和全民向心力，推动我市走上经济发展、生活富裕、生态良好的文明发展道路。

　　各位代表，今后五年的目标是宏伟的，任务是艰巨的。只要我们坚定信心，振奋精神，与时俱进，奋发有为，就一定能够顺利完成这些目标任务，推动全市综合实力、社会发展和人民生活登上新的台阶！

　　　　　　　　　　　　2003年的工作任务

　　2003年是新一届政府的开局之年，是迈出全面建设小康社会新步伐的重要一年。今年工作的总体要求是：以邓小平理论和三个代表重要思想为指导，深入贯彻党的十六大和省委十届三次、四次、市委九届二次全会精神，紧紧围绕全面建设小康社会和富民强市、快速崛起的奋斗目标，以加快发展为主题，继续实施开放兴市、科教兴市、以港兴市三大主战略，加快体制创新和科技创新，推进经济结构战略性调整，大力培育新的经济增长点，不断改善城乡人民生活，协调推进三个文明建设，全力维护社会稳定，实现经济持续快速健康发展和社会全面进步。

　　2003年国民经济和社会发展的主要预期目标是：国内生产总值增长11.5％，财政收入增长12.5％，全社会固定资产投资增长15％，社会消费品零售总额增长10％，外贸出口增长12％，直接利用外资增长25％，城镇居民人均可支配收入增长8％，农民人均纯收入增长8％，城镇登记失业率控制在4.5％以内，人口自然增长率控制在6‰以内，城镇化率达到34.5％。

　　围绕上述目标和要求，着重做好以下六个方面工作：

　　一、重抓经济结构调整，加快提升产业竞争能力

　　加快推进农业结构战略性调整，积极发展优质、高产、高效、生态、安全农业，提高农业综合生产能力和现代化水平，促进农业增效农民增收。一是大力发展区域特色农业。突出抓好特色园区、特色基地、特色乡镇建设，切实推进区域化布局和规模化生产。扩大蔬菜花卉、经济林果、牧草养殖、优质专用粮、水产育苗等特色产业规模，进一步发展黄川草莓、黑林板栗等特色生产基地，加快发展灌云优质棉和灌南特色林木，海淡水养殖面积达到106万亩。二是强力推进农业产业化经营。大力发展农产品深加工，扶持销售收入过千万元的109家规模龙头企业做大做强，10家省级重点龙头企业销售收入、利税增长15％以上。广泛招引三资开发农业，农产品出口创汇增长12％。三是切实抓好农产品质量安全管理。加快农产品质量安全标准与国际接轨，争创农业特色品牌，无公害农产品基地发展到100万亩，创无公害农产品品牌43个、绿色食品品牌12个。四是改善农业生产条件，健全社会化服务体系。加快农村道路、水电、通讯网建设，加强农业资源综合开发，提升农业机械化水平，广泛开展科技下乡、智力支农活动，推进农业信息进村入户，发展农村合作经济组织，壮大农产品运销大户，加强农民经纪人队伍建设，进一步搞活农产品流通。加大信贷支农力度，改善农村金融服务。

　　走新型工业化道路，着力调整、优化、提升产业结构，加快发展高新技术产业，促进产业结构优化升级，强化工业经济主体地位。一是采取资源整合、资本运作等多种措施，加快培育具有核心竞争力的大型企业集团，依托恒瑞、德邦等骨干企业壮大医药、化工、机械等优势行业，22户重点企业销售收入占工业比重提高2个百分点。二是强化项目建设，推进新产品开发，完成技术改造投资60亿元，竣工投产20个重点项目，开工建设30个重点项目，实施500万元以上工业技改项目170项，开发市级以上新产品250项，形成一批拥有自主知识产权、技术含量高、市场前景好的新产品，新产品产值率、销售率达26％以上。三是大力发展新医药、新材料、电子信息、机电一体化四大特色高新技术产业，壮大863成果产业化基地和新医药产业基地，实施抗肿瘤新药、新型中药、纺织后整理等一批示范工程，加快建设海洋化工、硅资源深加工等特色产业基地，高新技术产业增加值占工业增加值的比重提高到19％。四是促进高新技术改造传统产业，大力推进企业信息化，实施15项信息化带动工业化重点示范项目，推动企业广泛应用计算机辅助设计、企业资源计划等信息技术。抓好企业工程技术中心和博士后工作站的研究开发，提高企业核心竞争力。进一步做大建筑产业，加快企业改制步伐，全方位开拓建筑市场，建筑业增加值增长17.5％。

　　加快发展服务业，努力提高第三产业在国民经济中的比重。一是把发展港口经济作为振兴二、三产业的突破口来抓。修编港口发展总体规划，借鉴国际海港经验，实施港口管理体制改革。培育多元投资主体，加大投入，掀起港口建设的新高潮。建成7-10万吨级深水泊位，加快庙岭三期集装箱顺岸泊位和墟沟港区航道扩建工程建设。拓展港口综合功能，保持港口生产增长势头，完成港口吞吐量3500万吨，集装箱吞吐量25万标箱。积极启动以氧化铝、散化肥为突破口的物流服务，加快构筑以港口为龙头，以国际商务中心为平台，以数字化口岸和电子商务为支撑，以出口加工区、物流园区、开发区为依托，水路、铁路、公路、航空运输网络为骨架的现代物流中心。推进港口项目融资市场化、产业发展多元化、口岸服务信息化，提升港口综合竞争能力。二是做大做强旅游经济。积极创建中国优秀旅游城市，加快花果山、连岛、渔湾、孔望山、桃花涧等景区配套设施建设，继续办好连云港之夏、西游记文化旅游节等重大活动，精心培育旅游品牌。组织开展连云港旅游陆桥万里行活动，强化旅游资源经营者、导游队伍建设，综合整治旅游环境。推进旅游资源资本化运作、旅游项目市场化开发，提高旅游产品的开发水平与制造质量，拉长旅游产业链，国内外游客接待量突破400万人次，旅游总收入突破30亿元。三是着力提升商贸流通业。创新、整合经营业态，延伸发展专业店、专卖店、货仓式大卖场，以社区为中心发展多功能超市和连锁便利店，引导城镇超市等新型业态向农村拓展。加快改造传统商业，充分利用工商企业闲置设施，建设一批功能完备的社会化、专业化商品配送中心。完成社会消费品零售总额124亿元。四是加快发展房地产、社区服务等现代服务业。继续推进房地产业健康发展，竣工住宅面积95万平方米，完善房地产交易市场，满足多样化的住房消费需求，加强对房屋装饰装修和房屋安全管理。推进物业管理市场化，完善社区服务功能，扩大服务覆盖面，创建管理有序、文明祥和、安全便利的新型社区。

　　加快发展税源经济，建立稳定的财政增收机制。大力扶持主体税源和新型财源，振兴和壮大重点工业企业骨干财源；培育和拓展高新技术产业和私营个体经济新兴财源；以加快发展优质农产品和龙头企业为重点，稳固和强化农业基础财源；以县乡财政三年基本实现良性运行为目标，致力改善县乡财政困难状况，建立具有较强实力的地方财政。强化财税征管，确保应收尽收，提高财政收入占GDP的比重。调整优化财政支出结构，规范财政资金供给范围，保证重点支出需要。完善金融服务，突出信贷支持重点，扩大有效信贷投入，实现经济与金融协调发展。

　　二、深入推进各项改革，有效激发经济发展活力

　　加快推进国有企业改革，基本完成市属企业改制任务，重点在产权多元化和分配制度改革上取得突破。抓好正大天晴、港口股份上市争取工作，做好德邦兴华、豪森制药、中电华威上市辅导工作，推进连众等骨干企业的股份制改造。加快国有资本从一般竞争领域和中小企业基本退出，重点抓好40个改制项目，90％以上中小企业国有资本改制退出，按股比规范行使出资者权利。完善国有资产监管营运体系，实施分级管理、分级运营，促进国有资产保值增值。依托优势企业盘活存量资产，重组元升公司等8户破产企业，劣势企业基本退出市场。推动各类生产要素向优势产业、优质企业和优秀企业家集中。扩大国有企业改革领域，改善外经贸、旅游、商贸流通、交通运输、公用事业等行业的产权结构。

　　充分释放私营个体经济巨大增长潜力。把私营个体经济作为推进富民强市、改善财政状况、增加社会就业的有效途径和现实增长点，在市场准入、金融支持、税费征收、人才引进等方面制定和落实鼓励政策，坚定不移地营造加快发展的宽松环境。积极引导私营企业参与国有、集体企业改制改组改造，重点支持一批私营企业通过低成本扩张做大做强。规划建设好民营经济园区，积极培育各类要素市场，为私营个体经济发展提供有效载体和市场容量。新发展私营企业1500户、个体工商户1.2万户，新增从业人员3.5万人，非公有制经济占GDP比重提高3个百分点。

　　全面推进各项配套改革。一是深化投融资体制改革，推进市政公用设施由单纯政府投入向市场投入转变。按照经营城市的理念，运作好资产经营公司、城市建设投资公司、土地储备中心等发展主体。二是积极搞好农村三项改革，巩固农村税费改革成果，把减轻农民负担的政策措施落实好；按照依法、自愿、有偿的原则，建立市场化的土地承包经营权流转机制，发展规模经营；深化集体经济组织改革，推进机制和技术创新，提高乡镇企业的竞争能力。三是按照政事分开原则，推进事业单位分类改革，科学界定职能，理顺管理体制。四是继续推进财税改革，加强公共财政建设，深化预算编制改革，市级全面推行部门预算，加快实施国库集中收付制度，扩大政府采购规模和范围。五是深化行政审批制度和城市管理体制改革，进一步提高审批效率，相对集中一部分部门行政处罚权，组建新型高效的城市管理行政执法队伍。

　　三、突出抓好招商引资，全面提高对外开放水平

　　积极应对加入WTO和经济全球化的新形势，切实提升招商引资工作质量和水平，认真抓好投资环境、项目载体、招商效果三项重点工作，以改善投资环境作为培植项目载体的基础条件，以培植项目载体作为增强招商效果的有效手段，以增强招商效果作为提升招商水平的检验标准，政府推动、市场运作，实现环境招商、服务招商、功能招商、资源招商，推动全社会进一步形成浓厚的招商氛围。一是主攻招商重点，积极组织日韩、欧美等境外招商，大力推进东南沿海、陆桥沿线等国内招商，抓好日韩招商年活动，继续拓展对台招商，举办第五届江苏农业国际合作洽谈会。二是拓宽招商领域，重点发展工业招商，力求突破港口经济、城市建设招商，积极推动开发型农业、高新技术产业、商贸旅游业、现代服务业招商。三是培植项目载体，高度重视人才和物力两种投资承载体作用，通过尊重和发掘经济能人效用持续激发办项目热情，通过注重和强化招才引智有效拓展办项目空间，以优势产业和优强企业为龙头拉长产业链，以优势资源和特色园区为依托形成集聚效应。四是严格落实责任，层层分解目标，坚持两个月一次的项目过堂督查制度。强化激励机制，坚持奖惩兑现，继续对招商引资有突出贡献的单位和人员予以重奖。强化社会信用建设，打造诚信连云港。改善投资环境，进一步强化亲商、扶商意识，提高服务水平，营造优良的创业环境和人居环境。

　　加强各类开发园区的建设，合理布局，错位竞争，加快形成以国家级开发区为龙头、产业园区为特色、乡镇工业小区为支撑的发展新格局。营造良好环境，增强服务功能，强化园区综合竞争优势，不断提升园区的集聚、产出和创新功能，努力使园区成为招商引资的重要载体、产业转移的主要阵地和技术创新的有效平台。开发区在大项目招商和功能园区招商上实现重点突破，县区开发园区直接利用外资占到全市30％以上。落实共建共用开发园区的优惠政策，加快推动招商项目向园区集聚。确保赣榆海洋经济开发区获得省政府批准，千方百计争取出口加工区得到国家的支持，成为招商引资的新载体、新亮点。

　　抓住外贸经营权放开、经营主体扩大的机遇，积极壮大各类出口主体的规模和实力，巩固发展外贸企业和三资企业，加快培育出口生产企业，调整优化出口商品结构，保持外贸出口持续增长。加快口岸大通关建设，创造便利的商品进出口通道。积极实施走出去战略，提高外经工作层次和水平，规范外经劳务市场，扩大境外劳务输出，年末在外人数突破10000人。鼓励有实力的企业承揽海外承包工程，支持连众、康缘等企业到海外兴办实业。外经营业额增长12％。

　　四、强化基础设施建设，着力营造城乡发展环境

　　多渠道筹集资金，加强基础设施和综合环境建设，全社会固定资产投资突破200亿元。突出抓好水利、交通、能源、通讯、社会服务等42项重点基础设施项目。大浦闸拆建工程竣工，建成大浦河应急治理工程，尽快完成海堤达标工程，抓好小塔山水库除险加固，实施沭南大型灌区改造。建成连徐高速公路连云港段二期工程，开工建设连盐高速公路，改建304省道灌云南岗至燕尾段，创造条件争取改建213省道灌南段，继续实施干线公路网化和县乡村公路通达工程。完成民航候机楼改建。支持田湾核电站顺利建设，推动二期工程和抽水蓄能电站项目进程，开工500千伏连云港输变电、220千伏许庄输变电工程，续建四县县城电网改造、东海110千伏城北输变电工程，推进新电公司30万千瓦机组立项，发展新海地区集中供热，实施第二热电厂技术改造。以建设数字城市为目标，推进信息网络基础设施建设，建立网络化数字化信息资源体系，实施移动、联通公司移动电话和电信、网通公司程控电话扩容，加快建设干线传输网、接入网、用户网和基础地理信息系统，大力推进政府、企业、社会信息资源开发利用，完善金融、财税、社会保障等骨干信息系统，推进国民经济信息化。实施广播中波发射台搬迁，开工建设市博物馆和妇女儿童活动中心。

　　科学搞好城市近期建设规划，实现重点路段和重点区域详细规划全覆盖，精心设计城市标志性地段和标志性建筑，提高城市景观设计水平和空间环境质量，加强规划全过程管理，维护规划的严肃性。把拉开城市框架与完善城市功能有机结合起来，新浦区立足中心城区主体东移，连云区立足山海特色向西延伸，海州区立足古城风貌向东拓展，围绕新区开发、旧城改造、市政道路、配套设施、园林绿化、景区建设、美化亮化，投资30亿元实施140项城市基础设施项目。新区形成全面开发态势，重点构建路网框架，行政中心主楼主体工程封顶，房地产成片开发。旧城改造重点实施一批沿街地块和棚户区改造项目，改善群众居住质量。继续高标准构建城市道路网络，综合改造新港城大道凌洲广场至宋跳立交桥段，建设海宁中路、郁州北路、盐河南路、海滨大道等一批城市干道，打通龙河南路等7条便民道路，做好海宁路、新建路跨越盐河的桥梁建设准备工作。继续抓好公用基础设施建设，续建新海污水处理二期工程，筹建东部城区污水处理厂，建成危险废物处置中心，实施液化天然气改造工程，发展管道燃气用户4000户，建成人防工程9400平方米。以创建国家园林城市为目标，实施道路、河滨、广场、小区等园林绿化配套项目，新增城市绿地150万平方米。加大城市综合管理力度，加快实施重点路段、园区广场、窗口地区的美化亮化工程，塑造市容整洁、环境优美、秩序井然的城市新形象。

　　加快发展县城，重点择优发展中心城镇，构建现代化城镇体系。高起点建设县城，搞好市政配套，提高建设品位，完善交通环境，推进灌云、东海、赣榆污水处理厂和灌南中心路建设，加快建成交通便捷、功能齐全、环境优美的县域经济中心。以10个省级中心镇为重点，加快小城镇建设，增强小城镇在城乡经济社会发展体系中的纽带和牵引作用。

　　五、协调发展社会事业，不断改善人民生活质量

　　加快实施科教兴市战略，有效提高市民科学文化素质，强化人才智力支持，在全社会形成尊重劳动、尊重知识、尊重人才、尊重创造的良好氛围。普及科学知识，鼓励技术创新，弘扬科学精神。实施省级以上火炬计划项目20项、星火计划项目30项。加强专利和知识产权保护工作。发挥哲学社会科学在经济社会发展中的重要作用，加强对重大现实问题的调查研究。把教育摆在优先发展的战略地位，高质量发展教育事业，全面推进素质教育。健全社会化学前教育网络，继续巩固两基成果，高中阶段教育入学率提高到75％以上。进一步整合教育资源、改善办学条件，改造薄弱学校66所，新扩建中小学校舍20万平方米。提高地方高校办学层次，完善现代教育区建设规划。支持淮海工学院建设发展，积极推进江苏海洋大学的创建工作。大力开展远程教育和网络教育，建设教育城域网。积极发展职业教育和继续教育，延长新增劳动力受教育年限。建立培养与引进相结合的人才积累机制，形成广纳群贤、人尽其才的用人机制，鼓励知识、技术、管理等生产要素参与分配，创造良好的生活条件和创业环境。

　　加强精神文明建设，加快发展各项社会事业，不断增强可持续发展能力，使人民群众从发展中得到更多的精神文化享受。大力发展先进文化，以城市社区和农村乡镇为重点，以群众性文明创建活动为载体，繁荣社区文化、企业文化和校园文化，使全体市民始终保持健康向上的精神状态。创作一批反映时代特点和地方特色的文艺作品，重视文物保护工作。加快有线电视进村入户。完成东方医院门诊楼主体工程，建设市中医院门诊综合楼。广泛开展群众体育活动，组团参加第五届城运会，办好全国帆船、拔河锦标赛，申办全国十运会沙滩排球和武术散打比赛。抓好人口与计划生育工作，计划生育率达到89％以上。合理利用土地资源，加强矿产资源综合管理，继续抓好云台山植被恢复。实施蓝天、碧水、清洁、宁静四大环境整治工程和生态示范工程。加强国防教育，增强国防后备力量建设，依法推进城市民兵改革，搞好拥军优属、优抚安置，争创双拥模范城四连冠。搞好第三产业普查。建设黄海海域地震观测台。抓好外事、侨务和对台事务。做好档案、气象、地方志、新闻出版、民族宗教等工作。

　　千方百计扩大就业，持续增加城乡居民收入，不断完善社会保障体系，提高人民生活水平和质量。就业是民生之本，把增加就业岗位和开展就业援助作为各级政府的重要职责，把加快经济发展作为广开就业门路的根本举措，既积极发展高新技术产业，又继续发展劳动密集型产业；既积极发展大企业，又扶持发展中小企业；既加强国有集体企业改组改造，又大力发展非公有制经济。健全服务体系，落实就业政策，加大资金投入，注重解决特困群体再就业，继续推进政府出资购买就业岗位，全年实现城镇失业人员就业和再就业1.5万人。消除影响农村劳动力转移的政策障碍，多渠道转移农村富余劳动力。通过发展农村二三产业转移一批、搞活私营个体经济吸纳一批、推进城乡一体化融入一批，特别是通过扩大对内对外经济技术合作输出一批，力争每县新增劳务输出2万人，全市劳务输出达60万人。加强对农民的培训，开展农民培训年活动，年内培训15万人以上。在鼓励一部分人先富起来、扩大中等收入者比重的同时，注重提高低收入者的收入水平。支持企业发展促进在岗职工收入稳步增长，确保离退休人员基本养老金按时足额发放，保障失业人员基本生活。认真落实农村各项基本政策，切实防止农民负担反弹。抓好扶贫开发和老区建设，按照资金扶持、项目覆盖、干部帮扶、技术服务四到户的要求，做好农村困难群众救助帮扶工作，改善贫困户生产生活状况。注重关心弱势群体，切实维护妇女、未成年人、老年人、残疾人的合法权益。加快完善社会保障体系，继续做好两个确保和三条保障线的衔接工作，做好下岗失业人员生活保险关系的接续，养老、失业保险争取做到全覆盖，扩大困难群体参加医疗保险的比例。完善城市居民最低生活保障制度，对城镇生活困难较大的家庭，在就医、子女入学、住房等方面给予优惠和照顾。积极推行以大病统筹为主的新型农村合作医疗制度，新增农村改水受益人口16.5万人。强化政府救灾救济职能，建立和完善社会救灾、救济、互助、福利体系。高度重视安全生产，强化城乡公共安全管理，保护社会财产和人民生命安全。

　　六、切实加强自身建设，全力塑造政府良好形象

　　新一届政府肩负着人民的重托和光荣的使命，必须确立以人为本、执政为民的思想，以与时俱进、奋发有为的精神，加强服务型政府建设，奋力开创各项工作新局面。

　　加强民主法制建设，大力推进依法治市。坚持政府向人大及其常委会报告工作、向人民政协通报情况的制度，自觉接受监督。切实办好人大议案、人民代表建议和政协提案。听取各民主党派、人民团体和无党派人士的意见。完善了解民情、反映民意、集中民智、珍惜民力的决策机制，建立健全社会公示和专家咨询制度，推进决策科学化民主化。健全基层民主自治制度，完善村民自治和社区居民自治，坚持以职代会为基本形式的企事业单位民主管理制度，尊重和保障公民合法权益。提高政府工作法制水平，重视行政措施制定的公众参与度，加强行政执法监督，推进依法行政。深入开展四五普法教育，提高全民法律素质，增强公务人员法制观念和依法办事能力。

　　有效化解社会矛盾，坚决维护社会稳定。加强社会治安综合治理，提高治安防范控制能力，健全社会救援服务体系，创建安全稳定的社会环境。依法严厉打击各类犯罪活动，继续开展专项整治行动，防范和惩治邪教组织的犯罪活动，依法严惩骗税逃税、制假售假等违法犯罪行为，整顿和规范市场经济秩序，扫除社会丑恶现象。做好人民来信来访工作，健全人民调解工作机制，及时处置各类群体事件，全力维护社会稳定。

　　加快转变政府职能，不断改进干部作风。继续推进政府机构改革，科学规范部门职能，巩固软环境整治成果，加快形成行为规范、运转协调、公正透明、廉洁高效的行政管理体制。树立诚信政府形象，重在言必行、行必果：一是政府向人代会报告的工作坚持事事追踪，千方百计逐项完成；二是政府发文坚持少而精，做到令行禁止，贯彻到位；三是政府及组成部门向社会做出的各项承诺坚持一诺千金，说到做到。坚决防止和克服形式主义、官僚主义，坚持群众观点，强化公仆意识，保持与人民群众的血肉联系，始终把群众的利益放在心上，真心诚意、竭尽全力为群众多办实事。

　　继续加强廉政建设，牢固坚持执政为民。进一步抓好领导干部廉洁自律、查处大案要案、纠正部门和行业不正之风三项工作，标本兼治，不断加大从源头上预防和解决腐败问题的力度。认真落实党风廉政建设责任制，健全述职述廉、民主评议、重大事项报告和领导干部任期经济责任审计制度。按照党的要求和人民的期望，廉洁奉公，勤政为民，正确行使行政权力，以政府工作人员良好的形象取信于民。

　　各位代表，党的十六大描绘了中国特色社会主义事业的光辉前景。展望现代化建设的大好前程，我们豪情满怀；面对新一年改革发展稳定的艰巨任务，我们深感责任重大。让我们紧密地团结在以胡锦涛同志为总书记的党中央周围，高举邓小平理论伟大旗帜，全面贯彻三个代表重要思想，在中共连云港市委的领导下，坚持实干兴业，发展第一，团结和依靠全市人民，抢抓机遇，奋勇争先，与时俱进，开拓创新，为实现全面建设小康社会的宏伟目标而努力奋斗。
